# Time restricted EAting for Type 2 diabetes and MEtabolic health: The TEA TIME trial study protocol

**DOI:** 10.1371/journal.pone.0343494

**Published:** 2026-03-12

**Authors:** Matthew Retnakaran, Vittória L. Lessa, Caroline K. Kramer

**Affiliations:** 1 Leadership Sinai Centre for Diabetes, Mount Sinai Hospital, Toronto, Canada; 2 University of Vila Velha, Espirito Santo, Brazil; 3 Division of Endocrinology, University of Toronto, Toronto, Canada; 4 Lunenfeld-Tanenbaum Research Institute, Mount Sinai Hospital, Toronto, Canada; PLOS: Public Library of Science, UNITED KINGDOM OF GREAT BRITAIN AND NORTHERN IRELAND

## Abstract

**Background:**

Recent findings have suggested that implementing the emerging weight-loss strategy of time-restricted eating (TRE) for 6 weeks can have beneficial effects on the key pathophysiologic determinants of type 2 diabetes (T2DM) – namely, pancreatic beta-cell function and insulin resistance. Given the chronic nature of T2DM and the general challenge of long-term adherence to dietary interventions, a critical question is the durability of such effects. Specifically, we seek to determine whether TRE can improve the metabolic health of patients with T2DM over 1 year.

**Methods:**

In this open-label, parallel-arm, randomized controlled trial, individuals with overweight/obesity and T2DM of <10 years duration will be randomized to either standard lifestyle recommendations or TRE. The TRE protocol will consist of 18 hours of fasting and a 6 hour window of eating (between 2–8 PM) each day. The duration of the intervention will be 52-weeks and participants will undergo metabolic characterization at baseline, 12–24-, 36- and 52-weeks. The primary outcome of pancreatic beta-cell function will be assessed by Insulin Secretion-Sensitivity Index-2 (ISSI-2). Additional metabolic measures will include insulin resistance and glucose homeostasis.

**Discussion:**

TRE may represent an adjunct therapeutic approach for improving the metabolic profile of overweight/obese individuals with T2DM while also providing the capacity for modification of its underlying pathophysiology. This evaluation of the durability of the metabolic effects of TRE on beta-cell function and insulin resistance could thus identify a role for this dietary strategy as a disease-modifying intervention early in the course of T2DM.

**Trial registration:**

clinicaltrials.gov NCT07272460.

## Introduction

### Background and Rationale

The relationship between excess weight and type 2 diabetes (T2DM) is clear with approximately 61% to 74% of T2DM cases directly attributable to obesity [1–3]. Excess weight confers additional morbidity as increased BMI in these patients has been linked to poorer cardiovascular risk profile and higher mortality [4–7]. In this context, nutritional therapies targeting weight loss have been shown to improve glycemic control and, in turn, reduce the need for glucose-lowering medications in patients with T2DM [8–10]. However, studies of lifestyle interventions in T2DM have reflected the challenges of maintaining weight loss and achieving sustained glycemic control using non-pharmacological approaches [11,12]. Indeed, a meta-analysis of eleven such trials came to the conclusion that there was no significant impact on glycemic control after one year [11]. The long-term challenges of lifestyle changes in this patient population likely reflect not only the difficulty of prolonged adherence to lifestyle modification but the progressive natural history of T2DM. The natural history of T2DM is driven by 2 main pathophysiologic defects: (1) target cell resistance to the activity of insulin (insulin resistance) and (2) insufficient secretion of insulin by the pancreatic beta-cells to compensate for this peripheral tissue resistance (beta-cell dysfunction) [13,14]. The chronic nature of T2DM is driven by the progressive worsening of beta-cell dysfunction over time, which itself can be further exacerbated by insulin resistance [15]. While lifestyle interventions that have achieved weight loss have been shown to improve insulin resistance [3,9], their impact on pancreatic beta-cell function is not fully understood. Overall, there is no current anti-diabetic medication or lifestyle intervention that has conclusively been shown to prevent the deterioration of beta-cell function in patients with T2DM [14]. Thus, a fundamental problem in the clinical management of T2DM is the inability of current interventions to prevent the deterioration of beta-cell function that underlies the progressive natural history of this condition [13,14].

A recent lifestyle intervention of interest is time-restricted eating (TRE), which involves limiting food intake to a prescribed window of 4−8 hours [16–20]. Unlike traditional diets, TRE does not require calorie counting or specific food regulations/restrictions yet can achieve weight loss comparable to standard calorie-restrictive approaches [21,22]. Beyond weight reduction, TRE can offer additional metabolic benefits by promoting a shift from glucose utilization via glycogenolysis to lipolysis during prolonged fasting which is a pathway less dependent on insulin [23]. This shift suggests TRE may be a particularly relevant tool in managing conditions characterized by hyperinsulinemia (such as insulin resistance). Given its implications for insulin resistance, the impact of TRE on beta-cell function is of interest. To address this question, we recently conducted a randomized cross-over trial in which 39 overweight/obese participants with early T2DM completed 6 weeks of TRE (20 hours fasting/ 4 hours eating) or standard lifestyle [24]. Compared to standard lifestyle, TRE improved beta-cell function (Insulin Secretion-Sensitivity Index-2 (ISSI-2)), reduced hepatic insulin resistance (Homeostasis Model Assessment of Insulin Resistance (HOMA-IR)), and lowered HbA1c (−0.32 ± 0.48%, p < 0.001) [24]. These improvements were accompanied by reductions of body weight (−3.86 ± 3.1%, p < 0.001), and waist circumference (−3.8 ± 7.5 cm, p = 0.003) [24]. The results of this pilot study suggest that TRE is a pragmatic lifestyle intervention that can improve beta-cell function, insulin resistance, and glucose metabolism in overweight patients with T2DM, accompanied by beneficial effects on adiposity and negligible adverse effects. However, the outstanding question that remains is whether this lifestyle intervention can be sustained over time [25]. We are thus performing an open-label, parallel-arm, randomized controlled trial to determine whether TRE over 52-weeks is associated with improvement in beta-cell function, insulin resistance, and glucose homeostasis as compared to standard lifestyle in overweight/obese individuals with early T2DM.

### Objectives and hypothesis

Our trial has 3 main objectives. Firstly, the primary objective in this study is to evaluate the impact of TRE on beta-cell function in overweight/obese individuals with early T2DM as compared to standard lifestyle recommendations over a 52-week period. Our secondary objective is to evaluate insulin resistance in people implementing TRE in comparison to those who are applying standard lifestyle intervention. Lastly, our tertiary objective is to evaluate the impact of TRE on glucose homeostasis as compared to standard lifestyle intervention. Our hypothesis is that 52 weeks of TRE will yield (i) improvement in pancreatic beta-cell function, (ii) reduction in insulin resistance, and (iii) improvements in glucose homeostasis as compared to standard lifestyle recommendations.

## Methods

### Trial design and study population

**Fig 1:**
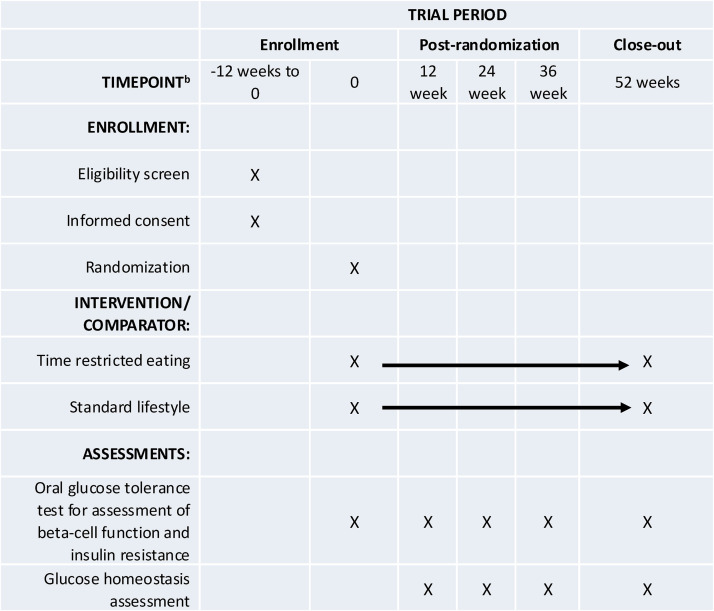
Participant timeline: Schedule of enrollment, interventions, and assessments.

**Fig 2:**
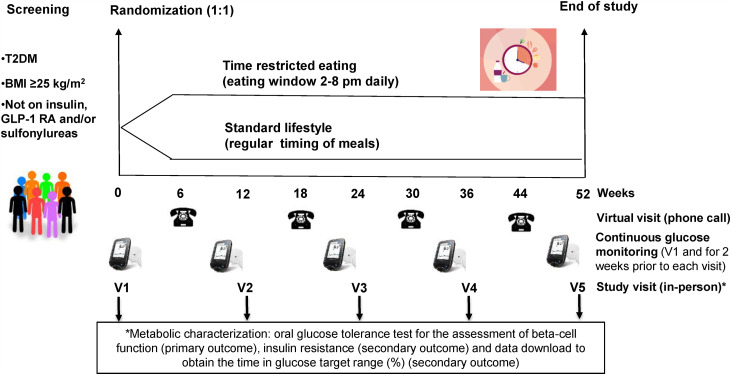
Schematic of study design.

This study is an open-label, parallel-arm, randomized controlled trial that will last a total of 52 weeks. A schematic of the study design can be found in Figs 1 and 2. In this trial, participants with overweight/obesity and T2DM will be randomized to either standard lifestyle recommendations or TRE. The inclusion and exclusion criteria for the study population are listed in Table 1. This study has just started recruitment and has estimated completion date of recruitment and follow up (data collection) of 31/July/2029 with completion of data analysis and reporting of results expected for 2030.

**Table 1:** Inclusion and exclusion criteria.

*Inclusion Criteria*
• Individuals with previously diagnosed BMI ≥ 25 kg/m^2^ and type 2 diabetes within preceding 10 years.
• Age 18–75 years inclusive
• Stable weight over past 12 weeks (less than 5% change in body weight) (self-reported)
• Diabetes treatment consisting of lifestyle only or metformin, dipeptidyl peptidase-4 (DPP-4) inhibitor, and sodium-glucose co-transporter 2 (SGLT2) inhibitors either as monotherapy or in combination.
• Ability to read and understand English
***Exclusion Criteria***
• Current diabetes treatment with insulin, glucagon-like peptide-1 receptor agonists, and/or sulfonylureas.
• Use of any other pharmacological treatment for weight loss
• Previous surgical treatment for weight loss such as gastric bypass or gastric band
• Any history of eating disorder
• Currently pregnant or lactating
• Renal dysfunction as evidenced by estimated glomerular filtration rate < 25 ml/min by CKD-EPI Creatinine Equation
• New York Heart Association class II-IV heart failure
• Hepatic disease considered to be clinically significant (includes jaundice, chronic hepatitis, or previous liver transplant) or transaminases > 2.5X the upper limit of normal
• Malignant neoplasm requiring chemotherapy, surgery, radiation or palliative therapy within the previous 5 years (with the exception of basal cell skin cancer)
• Any other factor likely to limit adherence to the study, in the opinion of the investigators
• Concurrent participation in another research study relevant to diabetes and metabolic health

### Randomization and intervention

Participants will be randomized 1:1 to either standard lifestyle recommendations or TRE protocol by random number generator (https://www.graphpad.com/quickcalcs/randomize1.cfm). Following randomization, the research coordinator will provide the participants with a booklet containing instruction on healthy eating as per Diabetes Canada Clinical Practice Guidelines [26,27] as well as answer any questions that arise. Participants will undergo metabolic characterization at weeks 0, 12, 24, 36, and 52. Those randomized to the standard group will be told to follow their normal schedule (e.g., possibly consisting of a 16 hour window for eating and ~8 hours of fasting daily). Individuals in the TRE protocol group will be instructed to only eat during a window of 6 hours (2–8 PM) while fasting for the remainder of the day. During the fasting period, participants will only be able to drink water, tea, and coffee (without any sugar added). Between the study visits, participants will be contacted by the research coordinator (virtual visits by phone) for reinforcement of the study protocol and assessment of safety. For 2 weeks after each study visit, participants will also wear an intermittently scanned continuous glucose monitoring system (isCGM) device (FreeStyle Libre 2) on their arms to monitor glucose levels. They will scan the isCGM at least 3 times a day, including before and after each meal. Participants will be instructed to record the time of each meal consumed during these two weeks using the FreeStyle Libre free app. Lastly, individuals who do not have a smartphone or who do not want to use their smartphone will be given a FreeStyle Libre 2 reader.

### Outcomes

The primary outcome will be beta-cell function at 52-weeks, measured by ISSI-2 [28,29]. The secondary outcomes will be insulin resistance at 52-weeks, measured by HOMA-IR [30], and percentage time in glucose target range at 52-weeks measured by FreeStyle Libre 2 [31]. Additionally, ancillary outcomes of interest will be comparisons between the study arms of the following measures at 52-weeks: (i) further measures of beta-cell function (ΔISR_0–120_/Δgluc_0–120_ × Matsuda index where ISR is the pre-hepatic insulin secretion rate determined by C-peptide deconvolution [32,33], insulinogenic index/HOMA-IR, and fasting pro-insulin/C-peptide ratio [34]), (ii) measures of insulin sensitivity (insulin sensitivity measured by Matsuda index on OGTT [35]), and (iii) anthropometric measures (body weight, body mass index (BMI), and waist circumference). We will also evaluate differences in measures of glucose homeostasis as assessed by isCGM (time above glucose target range (%), time below glucose target range (%), and mean glucose values), by OGTT (fasting glucose, 2-h glucose, area-under-curve of glucose from 0–120 min), and by HbA1c.

### Sample size

The primary outcome requires a sample size of 96 participants. This sample will provide 80% power to detect a 15% difference in ISSI-2 (%) between the study groups at a significant level (alpha) of 0.05. We performed this calculation using an estimated standard deviation of 18% as previously documented in our pilot study [24]. Factoring in an 18% loss-to-follow-up rate, a total of 112 participants, with 56 in each study group, would be required to ensure a minimum of 96 participants.

### Setting and recruitment

This trial will take place at a single academic center, Mount Sinai Hospital (MSH) in Toronto, Canada. It has received institutional research ethics and board approval (#REB 002) and all participants will provide informed consent form. We will conduct recruitment by contacting eligible participants at MSH, recruiting through nearby family physicians’ offices, and advertising with social media. To enable stratified analyses by sex, a maximum ratio of 1.5 female:male (n = 67 females and n = 45 males) will be recruited. Initial contact with interested participants will be made by one of the patient’s healthcare providers. Following the first interaction, the individuals will then be contacted by our research coordinator to determine eligibility. Potential participants will be emailed a consent form and provided with as much time as needed to review and decide on whether or not they wish to participate.

### Analysis plan

The primary analysis will be a comparison of the baseline-adjusted ISSI-2 between the study groups and will be evaluated by Students’ *t* test. In addition, given the repeated measures of metabolic characterization over follow-up, we will also evaluate the longitudinal change in ISSI-2 over time between the two study groups by Generalized Estimating Equation (GEE) model. The GEE model will examine (i) ISSI-2 change effect of TRE versus standard lifestyle, (ii) time effect on ISSI-2 changes, and (iii) treatment-by-time interaction (to determine whether ISSI-2 changes differentially over time between the study arms). Statistical analyses will only be performed after all study visits have been completed. No interim analysis is planned. Finally, given the sex differences in body fat deposition, we are planning stratified analyses by biological sex for the primary analysis.

### Knowledge dissemination

The knowledge translation plan includes methods that we will use to share the findings from this study. We will work with patient partners to ensure that the research results are communicated in a manner that is easily accessible to the patients’ community. Specifically, we will modify our knowledge dissemination strategies to target stakeholders. For researchers, we plan to make presentations at the American Diabetes Association (ADA) and Diabetes Canada annual meetings and the International Diabetes Federation (IDF) congress, as well as publish scientific papers. Patients will be informed about our findings at the end of the study through a newsletter sent out to all participants. Lastly, the general public will be informed of our findings through forms of media such as newspaper coverage, online articles, and television outlets.

No datasets were generated or analyzed during the current study. Data will be made available upon study completion.

## Conclusion and future perspectives

Lifestyle interventions are an important component of the multi-disciplinary clinical management of T2DM. Nonetheless, the long-term adherence to lifestyle interventions and the maintenance of resultant weight loss have proved to be challenging. Furthermore, lifestyle interventions have not been shown to affect the natural history of worsening beta-cell function over time in T2DM. If TRE is proven beneficial to metabolic health in overweight/obese individuals with T2DM over a prolonged period of time, these findings could ultimately impact nutritional recommendations in this patient population. A novel, economical, non-pharmacological intervention with the following three attributes could prove beneficial. These attributes would be the ability to induce weight loss, the capacity to improve glycemic control and, above all, the potential to improve beta-cell dysfunction. Thus, TRE might represent an adjunct therapeutic approach capable of improving the metabolic profile of overweight/obese individuals with T2DM while also providing the capacity for modification of its underlying pathophysiology that could ultimately change the natural history of diabetes.
